# Exploring solute binding proteins in *Pseudomonas aeruginosa* that bind to γ‐aminobutyrate and 5‐aminovalerate and their role in activating sensor kinases

**DOI:** 10.1002/mbo3.1415

**Published:** 2024-05-23

**Authors:** Jean Paul Cerna‐Vargas, Tino Krell

**Affiliations:** ^1^ Department of Biotechnology and Environmental Protection, Estación Experimental del Zaidín Consejo Superior de Investigaciones Científicas Granada Spain; ^2^ Centro de Biotecnología y Genómica de Plantas, Universidad Politécnica de Madrid Instituto Nacional de Investigación y Tecnología Agraria y Alimentaria/Consejo Superior de Investigaciones Científicas Parque Científico y Tecnológico de la Universidad Politécnica de Madrid, Pozuelo de Alarcón Madrid Spain

**Keywords:** molecular recognition, Pseudomonas aeruginosa, sensor histidine kinase, signal transduction, solute binding proteins

## Abstract

The standard method of receptor activation involves the binding of signals or signal‐loaded solute binding proteins (SBPs) to sensor domains. Many sensor histidine kinases (SHKs), which are activated by SBP binding, are encoded adjacent to their corresponding sbp gene. We examined three SBPs of *Pseudomonas aeruginosa* PAO1, encoded near the genes for the AgtS (PA0600) and AruS (PA4982) SHKs, to determine how common this arrangement is. Ligand screening and microcalorimetric studies revealed that the SBPs PA0602 and PA4985 preferentially bind to GABA (KD = 2.3 and 0.58 μM, respectively), followed by 5‐aminovalerate (KD = 30 and 1.6 μM, respectively) and ethanoldiamine (KD = 2.3 and 0.58 μM, respectively). In contrast, AgtB (PA0604) exclusively recognizes 5‐aminovaleric acid (KD = 2.9 μM). However, microcalorimetric titrations did not show any binding between the AgtS sensor domain and AgtB or PA0602, regardless of the presence of ligands. Similarly, bacterial two‐hybrid assays did not demonstrate an interaction between PA4985 and the AruS sensor domain. Therefore, sbp and shk genes located nearby are not always functionally linked. We previously identified PA0222 as a GABA‐specific SBP. The presence of three SBPs for GABA may be linked to GABA's role as a trigger for *P. aeruginosa* virulence.

## Graphical abstract

The protein AgtB has a specific affinity for 5‐aminovalerate. In contrast, both PA0602 and PA4985 show a strong preference for GABA, with secondary affinities for 5‐aminovalerate and ethylenediamine. Despite these findings, there is no evidence to suggest that these solute‐binding proteins—PA0602, PA4985, and AgtB—have any role in activating the AgtS and AruS sensor histidine kinases.

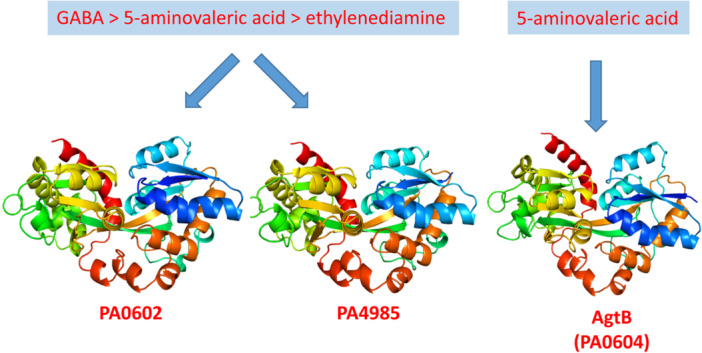

## 1 INTRODUCTION

The capacity of bacteria to adapt to changing environments is conferred by an array of different signal transduction pathways that either sense compounds in the cytosol or the extracytosolic space. Receptor families that permit extracytosolic signal recognition include sensor histidine kinases (SHK), chemoreceptors, adenylate, diadenylate, and diguanylate cyclases; cAMP, ‐di‐AMP, and c‐di‐GMP phosphodiesterases; and protein kinases and phosphatases that regulate different processes like gene expression, chemotaxis, second messenger levels or metabolism (Galperin, 2018; Gumerov et al., 2020).

The canonical receptor topology consists of an extracytoplasmic ligand binding domain (LBD) and a cytosolic output domain. Typically, a small molecule ligand interacts with LBD, generating a molecular stimulus that is transduced across the membrane where it triggers the generation of the signaling output. However, a major bottleneck in microbiology is the fact that the signal that stimulates the large majority of receptors, including systems that have been extensively studied, is unknown (Krell, 2015). This information is not only required to understand the corresponding regulatory circuit but also desirable to develop novel antibacterial strategies that are based on interference with bacterial signal transduction (Matilla & Krell, 2023; Plotniece et al., 2023). One of the reasons for this lack of information is the fact that receptors can also be stimulated by the binding of signal‐loaded solute‐binding proteins (SBPs) instead of direct ligand binding (Matilla et al., 2021).

SBPs form a super‐family composed of at least 33 individual Pfam families (Cerna‐Vargas et al., 2023). In Gram‐positive bacteria and archaea, SBPs are tethered to the external face of the membrane, whereas in Gram‐negative bacteria, they are present as diffusible proteins in the periplasm (Chu & Vogel, 2011). We have reported the SBP repertoire of 49 bacterial model strains that possess on average 60 ± 49 SBP genes, which frequently represent several percent of their genomes (Ortega et al., 2022). SBPs were shown to bind a wide range of ligands including amino‐ and organic acids, peptides, sugars, polyamines, quaternary amines, purines, metal ions, oxoanions, vitamins, quorum sensing signals and different siderophores (Cerna‐Vargas et al., 2023). In addition, a proteomics study has shown that the cellular levels of many SBPs are very high and generally superior to receptor levels (Matilla et al., 2023b). The high number of SBP genes and their elevated cellular levels reflect their physiological importance. The primary function of SBPs is to present different solutes to transporters to initiate their uptake (Davies et al., 2021; Elbourne et al., 2017). However, SBPs were also found to bind to receptors, permitting the coordination of transport with signal transduction (Matilla et al., 2021).

There are a number of observations that indicate that SBP‐mediated receptor activation is widespread in microorganisms. (i) Such mechanisms were shown to activate members of different receptor families like chemoreceptors (Lopes & Sourjik, 2018; Manson et al., 1986), SHKs (Moore & Hendrickson, 2012; Neiditch et al., 2005), protein kinases (Bhattacharyya et al., 2018) and diguanylate cyclases/phosphodiesterases (Sobe et al., 2017; Trimble & McCarter, 2011). (ii) The corresponding strains showed a wide phylogenetic distribution including α‐, β‐ γ‐ and ε‐ proteobacteria (Anderson et al., 2015; Antoine et al., 2005; Liu et al., 2012; Neiditch et al., 2006), firmicutes (Li et al., 2017) and archaea (Kokoeva, 2002). (iii) SBPs that belong to at least 13 different families were found to activate receptors, including members of the SBP_bac_8 family that will be studied here (Matilla et al., 2021). (iv) LBDs of all major families were found to bind SBPs (Matilla et al., 2021).

However, the identification of SBP–receptor interaction is technically not straightforward. For example, pull‐down assays can be conducted using immobilized LBD and cell extracts (Rico‐Jimenez et al., 2016). However, cellular SBP synthesis is tightly regulated (Matilla et al., 2021) and a given SBP may simply not be present in the cell extract. In addition, the detection of the SBP–LBD interaction often requires the signal molecule to be bound to the SBP (Matilla et al., 2021). However, for most of the systems analyzed, the signal is unknown, and the lack of the bound signal may account for the failure of pull‐down assays. Another issue that hampers the identification of SBP‐activated receptors is the fact that bacteria typically contain many SBPs which possess redundant ligand profiles (Ortega et al., 2022).

Interestingly, in 10 out of 11 characterized SBP‐SHK couples, we have noted that both genes are next to each other or in a very close vicinity (Matilla et al., 2021). Here, we aimed to investigate to what extent this is a general feature, which may be relevant for strategies aimed at a more rational identification of SBP‐stimulated receptors. To this end, we have chosen *Pseudomonas aeruginosa* PAO1 as a model, which is among the most feared human pathogens that cost the lives of more than half a million people each year (GBD, 2019 Antimicrobial Resistance Collaborators,19 Antimicrobial Resistance Collaborators, 2022). Strain PAO1 has 58 SHKs that establish two‐component systems (TCS) with 69 response regulators (Gumerov et al., 2023). Most *P. aeruginosa* TCS were found to regulate virulence or virulence‐related processes (Francis et al., 2017). However, the signal that stimulates most SHKs remains unknown (Francis et al., 2017).

**FIGURE 1:**
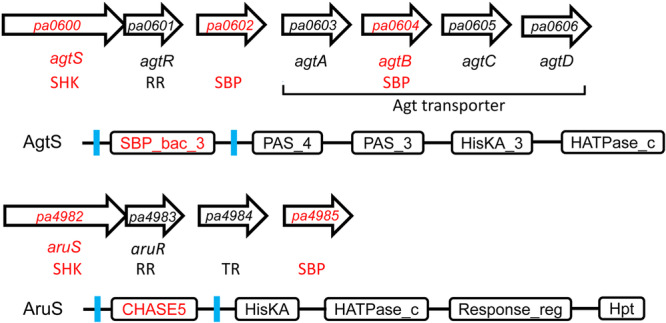
Organization of gene clusters encoding the AgtSR and AruSR two‐component systems and solute binding proteins (SBP). Domain arrangement of the AgtS and AruS sensor histidine kinases according to InterPro (Blum et al., 2021). Proteins analyzed in this study are shown in red. Transmembrane regions are in blue. RR, response regulator; SHK, sensor histidine kinase; TR, transcriptional regulator.

The inspection of the genetic neighborhood of all *P. aeruginosa* PAO1 SHKs showed that there are two cases in which TCS genes are in the vicinity of SBP genes. The first case is represented by genes *pa0600* to *pa0606*, which harbor the *agtSR* and *agtABCD* operons encoding a TCS and an ABC transporter, respectively (Chou et al., 2014) (Figure 1).

The *agtABCD* operon contains the *agtB* SBP gene. In between both operons is the *pa0602* gene encoding another SBP. This TCS‐transporter system permits the transport and growth of γ‐aminobutyrate (GABA) and 5‐aminovalerate (5‐AMV) (Chou et al., 2014). The *agtABCD* operon expression is induced by the exogenous addition of β‐alanine, GABA and 5‐AMV (Chou et al., 2014). Whereas mutation of each of the *agtSTABCD* genes abolishes growth on GABA and 5‐AMV, no such phenotypic change was observed for the PA0602 mutant (Chou et al., 2014). PA0602 is an uncharacterized protein, but a mutant showed reduced biofilm formation, susceptibility to tobramycin and increased transcript levels in biofilms (Kaleta et al., 2022).

The second case is the genes *pa4982* to *pa4985* encoding the AruSR TCS, a transcriptional regulator and the PA4985 SBP (Figure 1). The AruSR TCS controls the expression of the arginine transaminase pathway that catalyzes the conversion of l‐Arg into succinate (Yang & Lu, 2007). A phage display study has identified antigens that showed increased expression during early cystic fibrosis airway infections. Among the 76 antigens identified, the AruS SHK was the protein that appeared with the highest frequency, indicative of a role in infection (Beckmann et al., 2005). The molecular mechanism of AgtS and AruS SHK activation remains to be established. Furthermore, the ligands that bind to the SBPs PA0602, AgtB and PA4985 are also unknown but were predicted by TransportDB (Elbourne et al., 2017) to be spermidine and putrescine. These three proteins belong to the family SBP_bac_8 (Pf13416). This family was found to recognize primarily sugars but also polyamines, dinucleotides, Fe(III) and amino acids (Cerna‐Vargas et al., 2023). In this study, we aim to identify the ligands recognized by these three SBPs and assess their potential role in SHK activation.

## 2 MATERIALS AND METHODS

### 2.1 Bacterial strains and plasmids

Bacterial strains and plasmids used are listed in Table A1.

### 2.2 Protein overexpression and purification

Derivatives of expression plasmid pET28b(+) harboring the codon‐optimized sequences of SBPs PA0602, AgtB, and PA4985, as well as the LBDs of the SHKs AgtS (PA0600, amino acids 22–274) and AruS (PA4982, amino acids 31–141) were synthesized by GenScript. The AgtS and AruS LBDs correspond to the fragment in between the two transmembrane regions that were predicted using TMHMM (Krogh et al., 2001). The signal peptides of the SBPs were predicted using SignalP ‐ 5.0 (Almagro Armenteros et al., 2019) and were not included in the final protein. DNA fragments were cloned into the NdeI/HindIII sites of the vector that adds a His‐tag‐containing extension to the protein N‐terminus. A stop codon was introduced after the protein‐coding sequence to prevent the addition of a C‐terminal extension. The sequences of proteins analyzed in this study are shown in Table A2. *Escherichia coli* BL21 (DE3) cultures containing the expression plasmids were grown in 2L Erlenmeyer flasks containing 500 mL LB medium supplemented with 50 μg mL^−1^ kanamycin at 30°C until an OD_660_ of 0.6, at which point protein production was induced by adding 0.1 mM Isopropyl β‐d‐thiogalactopyranoside (IPTG). Growth was continued at 18°C overnight before cell harvest by centrifugation at 10,000 *g* for 30 min. Cell pellets were resuspended in buffer A (30 mM Tris, 300 mM NaCl, 10 mM imidazole and 5% (vol/vol) glycerol, pH 7.5) and broken by French press at 1000 psi. After centrifugation at 20,000 *g* for 1 h, the supernatant was loaded onto a 5 mL HisTrap column (Amersham Bioscience), washed with five column volumes of buffer A containing 45 mM imidazole and eluted with an imidazole gradient of 45–500 mM in buffer A. Freshly purified proteins were dialyzed overnight into TMP buffer (5 mM Tris‐HCl, 5 mM MES, 5 mM PIPES, 10% (vol/vol) glycerol, pH 8).

### 2.3 Thermal shift assays

The detailed experimental protocol of the thermal shift assays has been reported in (Fernandez et al., 2018). Briefly, assays were carried out using a MyIQ2 Real‐Time PCR instrument (BioRad). Experiments were conducted in 96‐well plates, and each assay mixture contained 20.5 μL of the dialyzed protein at 10–50 μM, 2 μL of 5X SYPRO orange (Life Technologies) and 2.5 μL of ligand solution or the equivalent amount of buffer in the ligand‐free control. Compound arrays PM1, PM2a, PM3B, PM4A, PM5, PM6, PM7, and PM8 from Biolog (Newark) were used. Compounds were dissolved in 50 μL water that, according to the information provided by the manufacturer, corresponds to a concentration of 10–20 mM. Samples were heated from 23°C to 85°C at a scan rate of 1°C/min. The protein unfolding curves were monitored by detecting changes in SYPRO Orange fluorescence. The Tm values correspond to the minima of the first derivatives of the raw fluorescence data.

### 2.4 Isothermal titration calorimetry (ITC)

Experiments were conducted on a VP‐microcalorimeter (Microcal). Proteins were dialyzed into the TMP buffer, placed at a concentration of 20–40 µM into the sample cell and titrated with 0.25–2.5 mM ligand solutions that were prepared using the dialysis buffer immediately before use. In the case no binding heats were observed in titrations with 14.42 μL aliquots of 10–20 mM ligand solution, it was concluded that there was no binding. The mean enthalpies measured from the injection of ligands into the buffer were subtracted from raw titration data before data analysis with the MicroCal version of ORIGIN. Data were fitted with the “One binding site model” of ORIGIN.

### 2.5 Thrombin cleavage

One milligram of protein dialyzed into the TMP buffer was incubated with one unit of thrombin (Merck) at 16°C for 1.5 h, at which point another unit of thrombin was added and incubated at 16°C for another 1.5 h. The protein solution was loaded onto a 5 mL HisTrap column (Amersham Bioscience) equilibrated with buffer A, washed with five column volumes of TMP buffer and eluted with an imidazole gradient of 45–500 mM in buffer A. Samples were analyzed by SDS‐PAGE. Fractions that correspond to cleaved protein were dialyzed overnight into the TMP buffer for ITC experiments.

### 2.6 BACTH assay

The bacterial two‐hybrid assay (BACTH) to monitor protein–‐protein interaction was conducted following the protocol of (Battesti & Bouveret, 2012). The DNA fragment corresponding to PA4985 (residues 40–363, signal peptide omitted) and the LBD of AruS (residues 31–141) were cloned in frame into pUT18C (resulting in pUT18C::PA4985) and pKNT25 (resulting in pKNT25::AruS). *E. coli* BTH101 was transformed with both plasmids, plated on LB supplemented with ampicillin, kanamycin, 0.1 mM of IPTG, 40 µg/mL X‐gal and when required 5 mM of GABA, 5‐AMV, spermidine or ethylenediamine. Plates were incubated at 30°C for 24–48 h. As a positive control, the plasmids pKT25‐zip and pUT18C‐zip were co‐transformed into *E. coli* BTH101. Negative controls were *E. coli* BTH101 harboring pUT18C and pKNT25::AruS (strain K^+^T^−^), and a strain harboring pUT18C::PA4985 and pKNT25 (strain K^−^T^+^).

### 2.7 Growth experiments

*P. aeruginosa* PAO1 was grown overnight in M9 minimal medium supplemented with 6 mg/L Fe‐citrate and trace elements (Abril et al., 1989). Cultures were washed twice and then diluted to an OD_600_ of 0.02 in M9 medium containing 10 mM ethylenediamine, spermidine, GABA, 5‐AMV or glucose (control) as the sole carbon source. Then, 200 μL of these cultures were transferred into microwell plates, and growth at 37°C was followed on a Bioscreen microbiological growth analyzer (Oy Growth Curves Ab Ltd.).

## 3 RESULTS

### 3.1 PA0602 binds preferentially GABA

**FIGURE 2:**
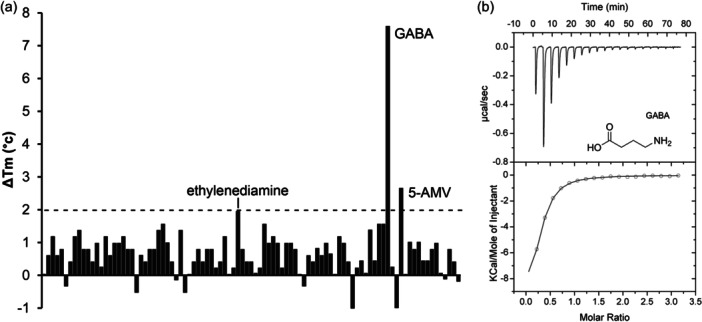
The PA0602 solute binding protein preferentially recognizes GABA. (a) Thermal shift assays using the compounds of array PM3B. The dashed line indicates the threshold that is considered significant for binding. 5‐AMV: 5‐aminovalerate. (b) Microcalorimetric studies of GABA binding by PA0602. Upper panel: the titration raw data for the injection of 4.8 µL aliquots of 1 mM GABA into 20 µM of PA0602. Lower panel: integrated, dilution heat corrected, and concentration normalized peak areas fitted with the “One binding site” model of ORIGIN.

The PA0602 protein was overexpressed in *E. coli* and purified. Freshly dialyzed protein was submitted to thermal shift assays‐based ligand screening using the Biolog compound arrays PM1, PM2A (both carbon sources), PM3B (nitrogen sources), PM4 (phosphorous and sulfur sources), PM5 (nutrient supplements), PM6, PM7, and PM8 (all peptide nitrogen sources). Ligand binding to the protein typically delays thermal unfolding causing increases in the midpoint of the protein unfolding transition (Tm). Tm increases of more than 2°C are considered significant. Screening of the corresponding 760 compounds resulted in significant Tm shifts for only GABA (7.6°C) and 5‐Amino valeric acid (5‐AMV, 2.3°C) (Figure 2a).

**FIGURE 3:**
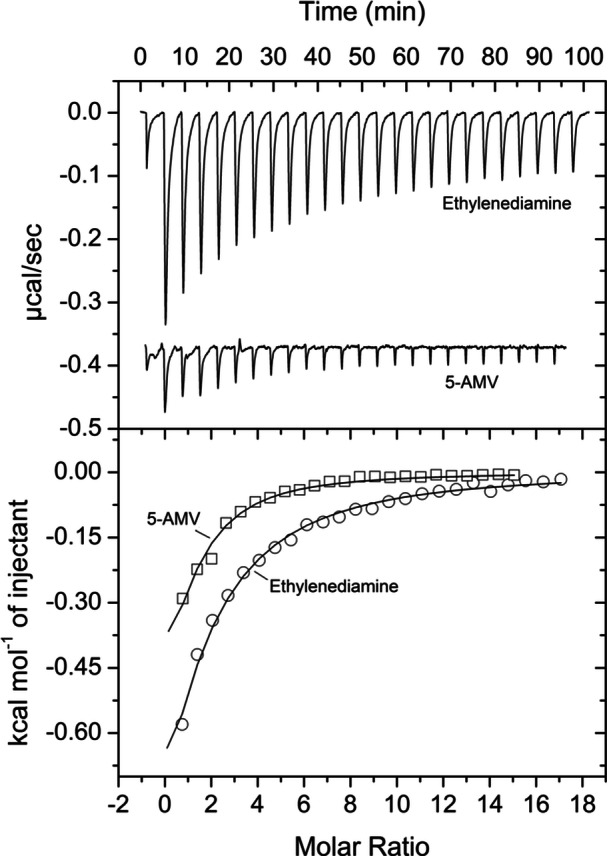
Microcalorimetric binding studies of 5‐AMV and ethylenediamine to PA0602. Upper panel: titration raw data for the injection of 11.2 µL aliquots of 2.5 mM ethylenediamine into 30 µM of PA0602 and 11.2 µL aliquots of 5‐AMV into 18 µM PA0602. Lower panel: integrated, dilution heat corrected, and concentration normalized peak areas fitted with the “One binding site” model of ORIGIN.

Microcalorimetric titration revealed that GABA bound with high affinity (*K*_D_ = 2.3 μM), whereas the affinity of 5‐AMV was lower (*K*_D_ = 30 μM) (Figures 2b and 3, Table 1). At times thermal shift assays provide false positive results, i.e. although there is binding, the Tm shift induced is only minor. Since PA0602 had been predicted by TransportDB to bind spermidine and putrescine, we also conducted microcalorimetric titrations with the polyamines spermidine, putrescine and ethylenediamine. Binding was solely observed for ethylenediamine that bound with an affinity significantly below that of GABA (*K*_D_ = 103 μM) (Figure 3).

**TABLE 1:** Summary of microcalorimetric ligand binding studies to solute binding proteins and histidine kinase sensor domains.

Protein	Pfam annotation	Binding studies		
		Ligand	Tm shift (°C)	*K*_D_ (µM)
PA0602	SBP_bac_8	GABA	7.6	2.3 ± 0.8
		5‐AMV	2.3	30 ± 2
		Ethylenediamine	2.0	103 ± 4
		Spermidine, putrescine, NADG	No binding	
AgtB	SBP_bac_8	5‐AMV	2.9	17 ± 0.8
		N‐acetyl‐D‐glucosamine, GABA, β‐alanine	No binding	
AgtS‐LBD	SBP_bac_3	No small ligand binding observed		
AgtS‐LBD		PA0602	No binding	
AgtS‐LBD		AgtB	No binding	
AgtS‐LBD/GABA^a^		PA0602/GABA	No binding	
AgtS‐LBD/5‐AMV^a^		AgtB/5‐AMV	No binding	
PA4985	SBP_bac_8	GABA	10	0.58 ± 0.1
		5‐AMV	7.6	1.6 ± 0.1
		Ethylenediamine	4.6	19 ± 1
		Spermidine	2.7	160 ± 23
		L‐Arg	No binding	
AruS‐LBD	CHASE 5	Insoluble protein		

^a^ These experiments were repeated with protein from which the His‐tag containing N‐terminal extension had been removed.

**FIGURE A1:**
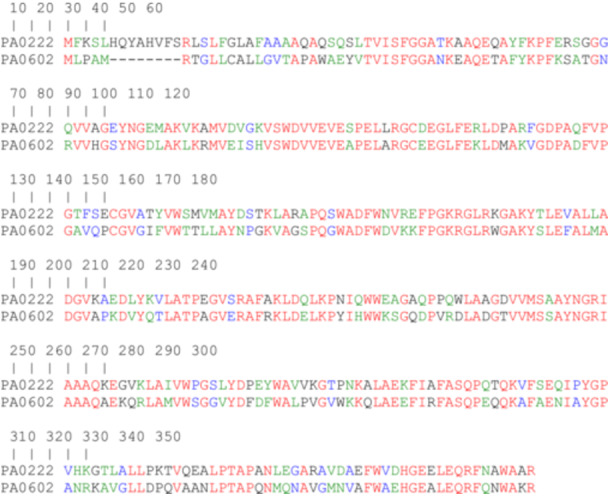
Sequence alignment of the PA0222 and PA0602 solute binding proteins. Both proteins share 55% identity (red), 20% strongly conserved amino acids (green) and 8% weakly conserved residues (blue). The alignment was done using the CLUSTALW algorithm of the npsa suite (Combet et al., 2000) using the GONNET weight matrix, a gap opening penalty of 10 and a gap extension penalty of 0.2.

With PA0222, we have previously identified a GABA‐specific SBP (Fernández et al., 2019). Both PA0602 and PA0222 are orphan proteins and their genes do not form part of transporter operons. Both proteins belong to the SBP_bac_8 (Pf13416) family and share 55% sequence identity (Figure A1).

### 3.2 AgtB binds specifically 5‐aminovaleric acid

**FIGURE 4:**
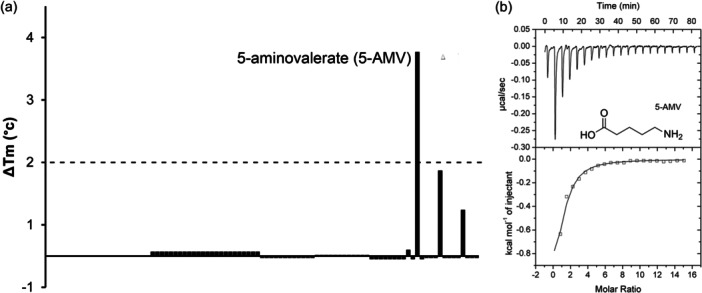
AgtB is a protein specific for 5‐aminovalerate. (a) Thermal shift assays using the compounds of array PM3B. The dashed line indicates the threshold that is considered significant for binding. (b) Microcalorimetric studies of 5‐aminovaleric acid (5‐AMV) binding to AgtB. Upper panel: titration raw data for the injection of 8 µL aliquots of 2.5 mM 5‐AMV ligand solution into 20 µM of AgtB. Lower panel: integrated, dilution heat corrected, and concentration normalized peak areas fitted with the “One binding site” model of ORIGIN.

**FIGURE A2:**
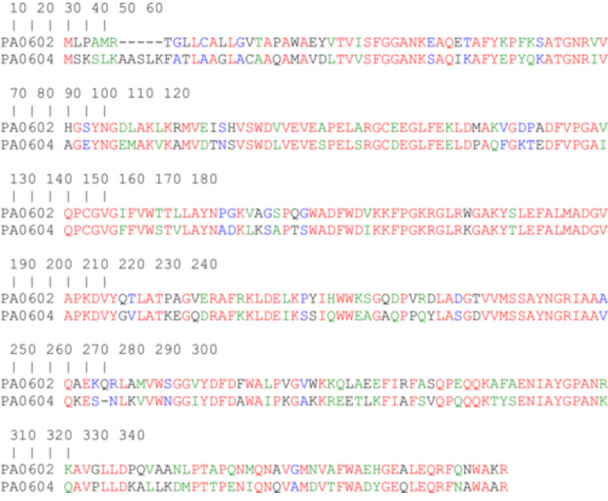
Sequence alignment of the PA0602 and PA0604 solute binding proteins. Both proteins share 60% identity (red), 17% strongly conserved amino acids (green) and 7% weakly conserved residues (blue). The alignment was done using the CLUSTALW algorithm of the npsa suite (Combet et al., 2000) using the GONNET weight matrix, a gap opening penalty of 10 and a gap extension penalty of 0.2.

The gene encoding the AgtB SBP (PA0604) is part of the *agt* transporter operon that is next to the *pa0602* gene (Figure 1). AgtB and PA0602 are also homologous and share 60% sequence identity (Figure A2). To identify the ligands that interact with AgtB, we pursued the same strategy and screened for ligand binding using the PM1 to PM8 compound arrays. 5‐AMV was the only compound that shifted the Tm by more than 2°C (Figure 4a).

Microcalorimetric titrations demonstrated that AgtB recognized 5‐AMV with a *K*_D_ of 17 μM (Figure 4b). Further titrations of the protein with GABA and the dipeptides Ala‐Gly and Gly‐Gln, which caused minor Tm shifts, did not reveal binding. AgtR was found to be required for N‐acetyl‐d‐glucosamine sensing (Korgaonkar et al., 2013). To assess whether this compound is a ligand of PA0602 and/or AgtB, we conducted thermal shift assays and microcalorimetric titrations that, however, indicated an absence of binding to both proteins. AgtB is thus a SBP specific for 5‐AMV.

### 3.3 Failure to identify ligands that interact with the AgtS‐LBD

**FIGURE A3:**
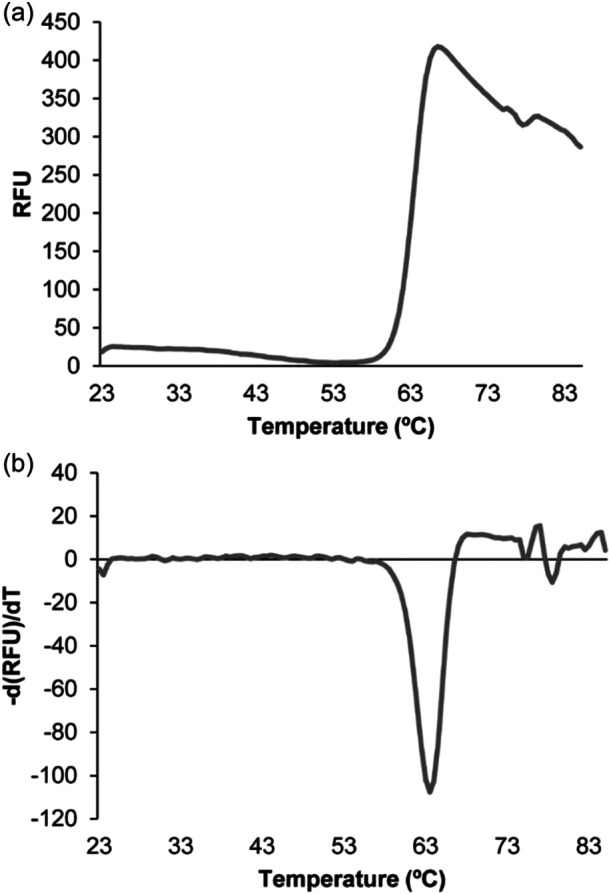
Thermal unfolding of AgtS‐LBD. (a) Raw unfolding curve. (b) First derivative of raw data. A Tm of 63°C was derived, indicative of protein folding.

The signals that activate the AgtS SHK are unknown. To address this issue, the individual AgtS‐LBD was produced as a purified, recombinant protein. A differential scanning fluorimetry analysis of the protein showed a very pronounced transition at a Tm of 63°C (Figure A3), indicative that the protein is folded. However, thermal shift assays of the protein using compound arrays PM1 to PM8 did not result in any significant shift. In addition, microcalorimetric titrations of AgtS‐LBD with N‐acetyl‐d‐glucosamine did not show binding.

To assess the possibility of SBP‐mediated activation of AgtS, we conducted protein‐protein microcalorimetric binding studies (Table 1). Five to 10 μM PA0602 or AgtB were placed into the instrument sample cell and titrated with 50–97 μM AgtS‐LBD, which, however, did not reveal binding. To explore the potential necessity of bound ligands to both SBPs for interaction with AgtS‐LBD, the experiments were repeated with samples that contained in both instrument compartments 5 mM 5‐AMV (for AgtB binding studies) or 1 mM GABA (for PA0602 binding studies). However, in none of the experiments, the heats observed were significantly different to those observed for the injection of AgtS‐LBD into buffer, indicating an absence of binding.

**FIGURE A4:**
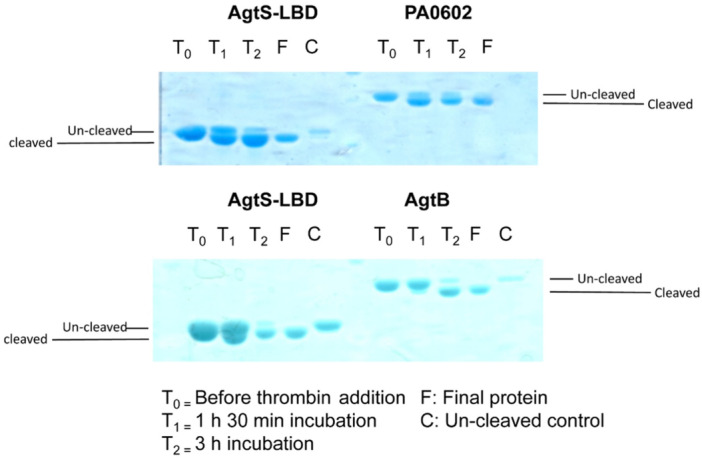
Removal of the N‐terminal tag in AgtS‐LBD, PA0602, and AgtB by thrombin cleavage. SDS‐PAGE gel of protein before cleavage, after 1.5 and 3 h and the final cleaved protein. As detailed in the “Materials and Methods” section, after an incubation of 3 h the protein was loaded onto a 5 mL HisTrap column. Whereas PA0600‐LBD and PA0604 eluted during the wash step using buffer A containing 45 mM imidazole, PA0602 was more tightly bound and eluted at the beginning of the imidazole gradient. Fractions were analyzed by SDS‐PAGE and cleaved proteins, labeled in this Figure as “F,” were used for experimentation.

Proteins used for these experiments contained an N‐terminal extension harboring the His‐tag. To exclude the possibility that the presence of this extension impedes protein interaction, the tag was cleaved with thrombin in freshly purified PA0602, AgtB, and AgtS‐LBD and its removal was verified by SDS‐PAGE (Figure A4). The above‐mentioned experiments in the presence of GABA and 5‐AMV were repeated with the His‐tag‐free protein, which, however, did not result in any protein–protein interaction.

### 3.4 PA4985 binds preferentially GABA and 5‐aminovalerate

**FIGURE 5:**
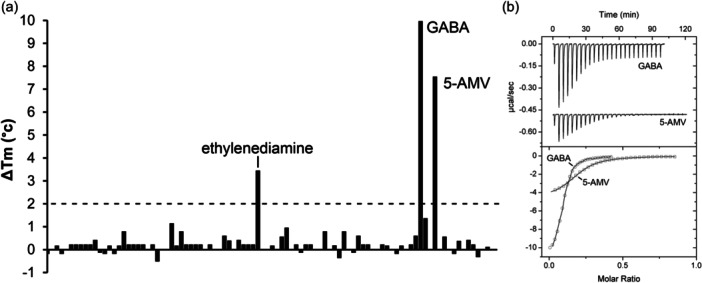
The PA4985 solute binding protein has a strong preference for GABA and 5‐aminovalerate. (a) Thermal shift assays using the compounds of array PM3B. The dashed line indicates the threshold that is considered significant for binding. (b) Microcalorimetric studies showing the binding of GABA and 5‐AMV (5‐aminovalerate) to PA4985. Upper panel: titration raw data for the injection of 4.8 µL aliquots of 0.25 mM GABA and 3.2 µL aliquots of 0.5 mM 5‐AMV into 40–50 µM of PA4985. Lower panel: integrated, dilution heat corrected, and concentration normalized peak areas fitted with the “One binding site” model of ORIGIN.

**FIGURE 6:**
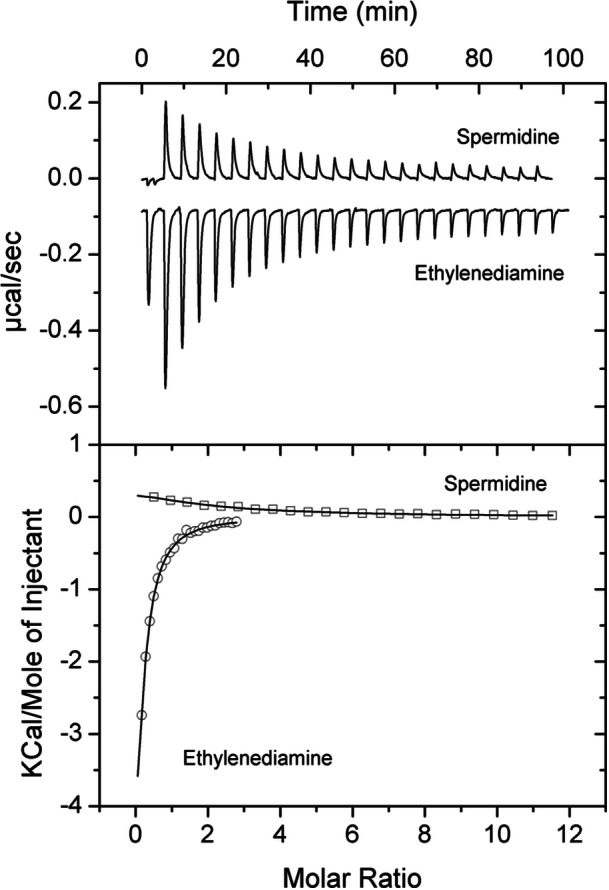
Microcalorimetric studies of the binding of spermidine and ethylenediamine to PA4985. Upper panel: raw data for the titration of 50 µM PA4985 with 12.8 or 3.2 µL aliquots of 2.5 mM spermidine and ethylenediamine, respectively. Lower panels: integrated, dilution heat corrected, and concentration normalized peak areas fitted with the “One binding site” model of ORIGIN.

PA4985 is an uncharacterized protein. Its gene is located in the vicinity of the *aruSR* TCS that controls the expression of the arginine transaminase pathway (Yang & Lu, 2007). Although encoded in a different part of the genome, PA4985 is homologous to PA0602 and AgtB, sharing 45% and 49% sequence identity with these proteins, respectively. The screening of PA4985 with compounds of arrays PM1 to PM8 resulted in substantial Tm shifts for GABA (10°C) and 5‐AMV (7.6°C), whereas more modest shifts were obtained for ethylenediamine (3.5°C) and spermidine (2.8°C) (Figure 5a). Microcalorimetric analyses showed that the magnitude of Tm increases correlated well with binding affinity. GABA bound most tightly (*K*_D_ = 0.58 μM), followed by 5‐AMV (*K*_D_ = 1.6 μM) (Figure 5b), ethylenediamine (*K*_D_ = 19 μM) and spermidine (*K*_D_ = 160 μM) (Figure 6).

### 3.5 Failure to observe an PA4985–AruS–LBD interaction

**FIGURE 7:**
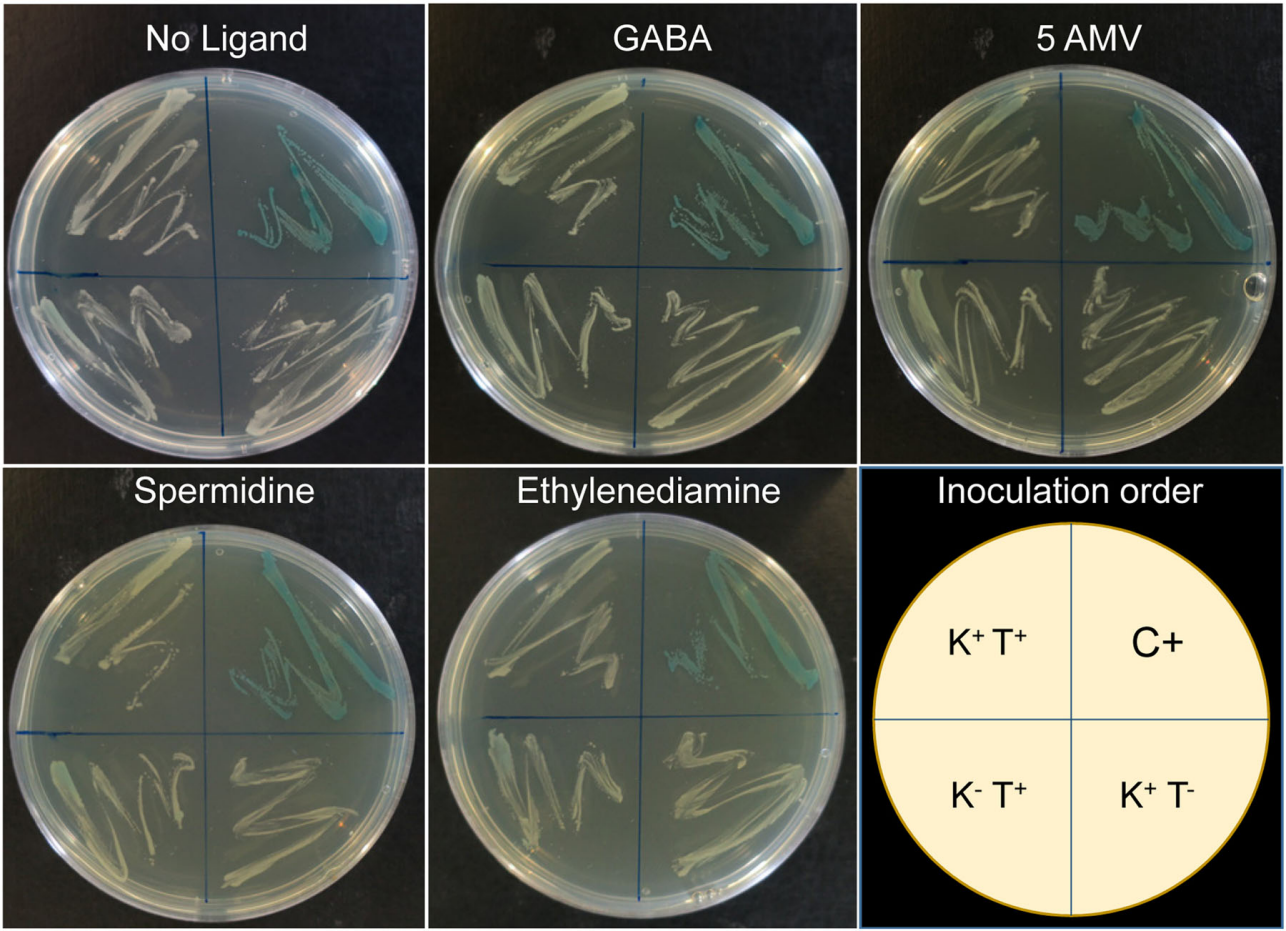
Bacterial two‐hybrid assay (BACTH) to monitor a possible interaction between PA4985 and AruS‐LBD. K^+^T^+^, encoding fusions of PA4985 and AruS‐LBD to adenylate cyclase subunits: C+, positive control; K^−^T^+^: negative control; K^+^T^−^, negative control. Further information on these strains is found in Table A1. Agar plates contained either no ligand or the ligands specified at 5 mM.

The LBD of AruS is relatively small (110 amino acids) and belongs to the family CHASE 5 (Figure 1). As with the other proteins in this study, the AruS‐LBD was overexpressed in *E. coli* and purified. However, despite extensive solvent engineering approaches, we failed to identify a buffer system that maintained protein solubility; a fact that prevented further in vitro studies. To monitor a potential PA4985 interaction with AruS‐LBD, we resorted thus to the bacterial two‐hybrid system‐based assay (BACTH) (Karimova et al., 2017). This system is based on an adenylate cyclase that contains two catalytically active domains. Only when both sub‐domains interact in an adenylate cyclase mutant *E. coli* strain (cya^−^, in this work BTH101), the adenylate cyclase activity is restored. The resulting increase in cAMP induces the expression of lactose and maltose operons, causing a blue colouration of bacteria on X‐Gal‐containing plates. We generated a plasmid that expresses PA4985 fused to one enzyme subunit (pUT18C::PA4985) and a plasmid encoding a fusion of AruS‐LBD to the other subunit (pKNT25::AruS). *E. coli* BTH101 was transformed with both plasmids, resulting in strain K^+^T^+^. The K^+^T^−^ and K^−^T^+^ are negative control strains that contained one of the plasmid constructs (plus sign) and the empty version of the second plasmid (minus sign). The positive control (C+) was provided by the manufacturer. These four strains were plated on X‐Gal‐containing plates that were either free of the ligand identified or that contained GABA, 5‐AMV, spermidine or ethylenediamine at a concentration of 5 mM. As shown in Figure 7, the positive control strain showed the expected blue coloring, whereas the remaining three strains were pale, indicating the absence of an PA4985/AruS–LBD interaction in the absence and presence of ligands.

### 3.6 Growth in the presence of GABA, 5‐AMV and spermidine

**FIGURE A5:**
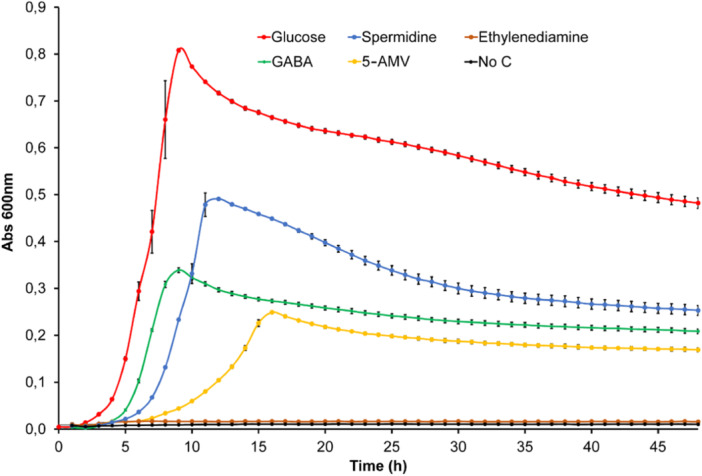
Growth experiments of *Pseudomonas aeruginosa* PAO1 in minimal medium without carbon source (No C) or in minimal medium supplemented with 10 mM of the compound indicated. Shown are means and standard deviations from three biological replicates.

To assess the metabolic value of the SBP ligands, we subsequently conducted growth experiments of *P. aeruginosa* PAO1 in minimal medium containing either glucose (positive control) or the ligands identified as the sole carbon source at a concentration of 10 mM. As shown in Figure A5, significant growth was observed in GABA, 5‐AMV and spermidine.

## 4 DISCUSSION

This study was inspired by the observation that in 10 out of 11 characterized examples of SBP‐activated SHKs, both genes were associated in the genome (Matilla et al., 2021). In contrast, in only 3 of 11 studied chemoreceptor/SBP couples, the chemoreceptor genes are next to SBP genes. Certainly, the genetic proximity of SBP and receptor genes suggests the possibility of functional associations. However, a significant number of SBP/receptor pairs have been identified by approaches like phenotypic screening of mutant libraries or pull‐down assays, that are not based on the genetic neighborhood (Matilla et al., 2021). Our data indicate that neither the SHKs AgtS nor AruS are activated by the three SBPs studied. This is thus consistent with the notion that an association of *shk* and *sbp* genes does not necessarily imply a functional relationship.

Our study has resulted in the definition of the ligand profile of three so far uncharacterized SBPs. All three proteins were predicted by TransportDB (Elbourne et al., 2017) to bind spermidine and putrescine. PA0602 and PA4985 are two orphan SBPs, i.e. not part of a transporter operon, that are encoded in different parts of the genome. Both proteins share 45% sequence identity. However, their ligand spectrum is almost identical (Table 1). Both proteins preferentially bind GABA, followed by 5‐AMV and ethylenediamine. In addition, we have previously shown that the SBP PA0222 binds exclusively to GABA (Fernández et al., 2019). All three SBPs bind GABA with high affinity as evidenced by *K*_D_ values of 0.58 μM (PA4985), 2.3 μM (PA0602) and 0.29 μM (PA0222) [Table 1, (Fernández et al., 2019)]. *P. aeruginosa* PAO1 has 93 SBPs, and bioinformatic predictions indicate a significant functional redundancy (Ortega et al., 2022). Our data provide experimental proof for this SBP redundancy, which remains poorly understood. Of the 93 SBPs, 62 SBPs were detected and quantified in a proteomics study. Among these proteins was only one of the three GABA‐responsive SBPs (PA0602) (Matilla et al., 2023b), whereas the remaining two proteins are likely to be present at levels below the mass spectrometric detection limit, indicating that the functional redundancy of genes does not necessarily translate into a redundancy of proteins.

GABA is a ubiquitous non‐proteinogenic amino acid that can be used by many bacteria as a carbon and nitrogen source (Zhu et al., 2020). GABA was shown to be a central signal molecule in all kingdoms of life. It is an important mammalian neurotransmitter, as well as a plant and bacterial signal molecule that controls a variety of processes (Caspi et al., 2023; Li et al., 2021; Quillin et al., 2021; Smart & Stephenson, 2019). Importantly, GABA was found to modulate the virulence of diverse pathogens. In the plant pathogen *Pseudomonas syringae* DC3000, GABA reduced the expression of type III secretion system genes (Park et al., 2010). Exposure of *P. aeruginosa* to 10 μM GABA did not modulate growth or motility but strongly increased cytotoxicity and virulence (Dagorn et al., 2013). In addition, biofilm formation, the production of adhesins and cyanogenesis were altered. The role of GABA in regulating virulence has been particularly well studied in *Agrobacterium tumefaciens*, where it controls the horizontal transfer of the Ti virulence plasmid (Lang & Faure, 2016; Lang et al., 2016), reduced the synthesis of quorum sensing signals (Chevrot et al., 2006; Dessaux & Faure, 2018; Wang et al., 2006) and cross‐talks to the salicylic acid and indole‐3‐acetic acid regulatory circuits (Yuan et al., 2008). The central role of GABA in *A. tumefaciens* also appears to be reflected in the redundancy of GABA‐responsive SBPs, since so far two GABA‐responsive SBPs have been identified, namely Atu2422 (Haudecoeur et al., 2009; Planamente et al., 2010) and Atu4243 (Planamente et al., 2012; Planamente et al., 2013).

SBP redundancy is also illustrated by the fact that all three SBPs bound 5‐AMV, which differs from GABA in only a methylene group. 5‐AMV is an intermediate of the lysine degradation pathway (Knorr et al., 2018). Next to the above‐mentioned PA0602 and PA4985, AgtB was identified as a specific 5‐AMV binding protein (*K*_D_ = 17 μM). The Agt transport system permits growth on 5‐AMV and GABA (Chou et al., 2014). As indicated in Figure 1, there are two SBP genes, *pa0602* and *agtB*, associated with the Agt transporter. Growth of the PA0602 mutant on both compounds was indistinguishable, whereas the AgtB mutant was unable to grow on 5‐AMV and was affected in its growth on GABA (Chou et al., 2014). Here we show that AgtB is a 5‐AMV‐specific protein, which does not entirely agree with the observation that its mutant has impaired growth on GABA. The identification of three SBPs for 5‐aminovalerate suggests a significant physiological relevance.

The physiological relevance of a given compound is frequently underlined by the evolution of specific chemoreceptors that mediate chemoattraction (Matilla et al., 2023a). Chemoreceptors for GABA have been identified in different *P. syringae* strains and receptor mutants showed reduced virulence (Santamaría‐Hernando et al., 2022; Tumewu et al., 2020). By analogy, *P. aeruginosa* PAO1 has a chemoreceptor, PctC, that preferentially binds GABA and mediates strong chemoattraction (Gavira et al., 2020; Reyes‐Darias et al., 2015; Rico‐Jiménez et al., 2013). Interestingly, the affinity of GABA for the PctC sensor domain (*K*_D_ = 1.2 μM) is in the range of that for the SBPs (Rico‐Jiménez et al., 2013). In analogy to GABA, the relevance of 5‐AMV is reflected by the fact that it is also recognized by the PctC chemoreceptor causing strong chemoattraction (Xu et al., 2023).

Further investigations are needed to establish to what degree multiple GABA and 5‐AMV binding SBPs are a more general phenomenon among bacteria. This knowledge in turn may help to identify the reasons and physiological relevance for the redundancy of SBPs that bind the same ligands.

## AUTHOR CONTRIBUTIONS

**Jean‐Paul Cerna‐Vargas**: Investigation; writing—review & editing; formal analysis; data curation. **Tino Krell**: Conceptualization; writing—original draft; writing—review & editing; funding acquisition; supervision.

## CONFLICT OF INTEREST STATEMENT

None declared.

## ETHICS STATEMENT

None required.

## Data Availability

The data that support the findings of this study are available in the article and its Appendix.

## 1

**TABLE A1:** Strains, plasmids and oligonucleotides used in this study.

	Relevant characteristics	Reference or source
Strains		
*Escherichia coli* BL21 (DE3)	F^–^ *ompT gal dcm lon hsdS*_*B*_(*r*_*B*_^–^ *m*_*B*_^–^) λ(DE3 [*lacI lacUV5*‐*T7p07 ind1 sam7 nin5*]) [*malB* ^+^]_K_^−^_12_(λ^S^)	Jeong et al. (2009)
*E. coli* BL21‐AI	F‐ *ompT hsdS*_B_ (r_B_^‐^m_B_^‐^) *gal dcm araB*::*T7RNAP‐tetA*	Invitrogen
*E. coli* DH5α	F^–^ *endA1 glnV44 thi‐1 recA1 relA1 gyrA96 deoR nupG purB20* φ80d*lacZ*ΔM15 Δ(*lacZYA‐argF*) U169, hsdR17(*r*_*K*_^–^ *m*_*K*_^+^), λ^–^	Woodcock et al. (1989)
*E. coli* BTH101	F‐, *cya‐99, araD139, galE15, galK16*, *rpsL1* (Str^R^), *hsdR2, mcrA1, mcrB1, relA1*	Euromedex
K^+^T^+^	BTH101 co‐transformed with pKNT25::PA4982 and pUT18C::PA4985 plasmids	This work
K^+^T^‐^	BTH101 co‐transformed with pKNT25::AruS and pUT18C plasmids, negative control	This work
K^‐^T^+^	BTH101 co‐transformed with pKNT25 and pUT18C::PA4985 plasmids, negative control	This work
C*+*	BTH101 co‐transformed with pKT25‐zip and pUT18C‐zip plasmids, positive control	This work
Plasmids		
pET28b(+)	Km^R^, protein expression plasmid	Novagen
pET28::AgtS‐LBD	Km^R^; pET28a(+) derivative containing DNA fragment encoding the LBD (residues 22 to 274) of PA0600	This work^a^
pET28::PA0602	Km^R^; pET28a(+) derivative containing DNA fragment encoding PA0602 (residues 22–344)^b^	This work^a^
pET28::AgtB	Km^R^; pET28a(+) derivative containing DNA fragment encoding AgtB (residues 27–348)	This work^a^
pET28::AruS‐LBD	Km^R^; pET28a(+) derivative containing DNA fragment encoding the LBD (residues 31–141) of PA4982	This work^a^
pET28::PA4985	Km^R^; pET28a(+) derivative containing DNA fragment encoding the LBD of PA4985 (residues 40–363)	This work^a^
pKNT25	Km^R^; p15A origin of replication. Designed to create in‐frame fusions at the N‐terminal end of the T25 protein	Euromedex
pKNT25::AruS	Km^R^; pKNT25 derivative containing DNA fragment encoding the LBD (31–141) of PA4982	This work
pUT18C	Ap^R^, ColE1 origin of replication. Designed to create in‐frame fusions at the C‐terminal end of T18 protein.	Euromedex
pUT18C::PA4985	Ap^R^, pUT18C derivative containing DNA fragment encoding PA4985	This work
pKT25‐zip	Km^R^, pKT25 derivative containing the leucine zipper region of the GCN4 yeast protein, positive control	Euromedex
pUT18C‐zip	Ap^R^, pUT18C derivative containing the leucine zipper region of the GCN4 yeast protein, positive control	Euromedex
Oligonucleotides		Restriction site (in bold)
Fw 4985 pUT18C	AAA**CTGCAG**ACCGGTCCAGGCCGATGAT	PstI
Rv 4985 pUT18C	TTT**GGATCC**TGCTGGCTGGCCCAGGCGT	BamHI
Fw 4982 pKNT25	AAA**GGATCC**CCAGCTGTTCTTCGAATACCG	BamHI
Rv 4982 pKNT25	TTT**GAATTC**GACAGCCGGCGATAGACCG	EcoRI

^a^ Plasmids synthesized by GenScript.

^b^ The signal peptide was not included.

**TABLE A2:** Sequences of proteins used in this study.

Name	Sequence
AgtS‐LBD	**MGSSHHHHHHSSGLVPRGSH**MESVALPLTAELRTWLAEHRELRVGVVTEAPYAQYDRRLQQYSGANVELMGWLATAMQVTLRWQGYPDQAALDDALRAGRVDIAPGLSQTPAGLRLWLFSDPYLRVSRLVVGKRDGSGSVELEQLERDTRVAVRAGSSVADYLSSTYSGLQWVSADSEREVLRAVLAGQARYAVIDEAQLSRLTREPEFAELAVVGDIGYPQLLRVATRRDWPELAMVVDQALRSLPRKSLDQLHERWLQPKYPRLGESPGFWQ
PA0602	**MGSSHHHHHHSSGLVPRGSH**MAEYVTVISFGGANKEAQETAFYKPFKSATGNRVVHGSYNGDLAKLKRMVEISHVSWDVVEVEAPELARGCEEGLFEKLDMAKVGDPADFVPGAVQPCGVGIFVWTTLLAYNPGKVAGSPQGWADFWDVKKFPGKRGLRWGAKYSLEFALMADGVAPKDVYQTLATPAGVERAFRKLDELKPYIHWWKSGQDPVRDLADGTVVMSSAYNGRIAAAQAEKQRLAMVWSGGVYDFDFWALPVGVWKKQLAEEFIRFASQPEQQKAFAENIAYGPANRKAVGLLDPQVAANLPTAPQNMQNAVGMNVAFWAEHGEALEQRFQNWAKR
PA0604	**MGSSHHHHHHSSGLVPRGSH**MAVDLTVVSFGGANKSAQIKAFYEPYQKATGNRIVAGEYNGEMAKVKAMVDTNSVSWDLVEVESPELSRGCDEGLFEELDPAQFGKTEDFVPGAIQPCGVGFFVWSTVLAYNADKLKSAPTSWADFWDIKKFPGKRGLRKGAKYTLEFALMADGVAPKDVYGVLATKEGQDRAFKKLDEIKSSIQWWEAGAQPPQYLASGDVVMSSAYNGRIAAVQKESNLKVVWNGGIYDFDAWAIPKGAKKREETLKFIAFSVQPQQQKTYSENIAYGPANKQAVPLLDKALLKDMPTTPENIQNQVAMDVTFWADYGEQLEQRFNAWAAR
AruS‐LBD	**MGSSHHHHHHSSGLVPRGSH**MQLFFEYRREMREIEARLELIRSGYLASFERSLWDLNQEQLNVQLRGLGDFPDIARVSLQSADFNLLQGDQRPRGMLRVERFPLSYQPPGGERRQLGELEIAIDLAAVYRRL
PA4985	**MGSSHHHHHHSSGLVPRGSH**MADDGLVVVGYGGAGQKAQEVAFFQPFTQQTGIPVVQSEYNGEMARIKVMADTGHADWDLVQVEGPDLARGCDDGLFERLDWQAIGGKQQLIANAAQECGSAALVWGVAIGYDADRLQQPPASWADFWDVQRFPGKRGLRKRAIYNLEFALLADGVPREQVYPLLATRAGADRAFAKLGQLKPYIQWWEAGAQPAQWLAAGDVVMTSTYTGRIADAHRAGRNLALVWQGSLYGMDYWAVVKGSKRGAEARRFIAFANAPDAQVRYVENIPYGPTNRQAAQRLPAQLASWVPTAPANLEQGLAMDDEFWVEHGEELEERFNAWASQ

*Note*: The His‐tag containing extension is in boldface, and the thrombin cleavage site is underlined.
